# Author Correction: Deciphering driver regulators of cell fate decisions from single-cell transcriptomics data with CEFCON

**DOI:** 10.1038/s41467-024-45929-1

**Published:** 2024-02-13

**Authors:** Peizhuo Wang, Xiao Wen, Han Li, Peng Lang, Shuya Li, Yipin Lei, Hantao Shu, Lin Gao, Dan Zhao, Jianyang Zeng

**Affiliations:** 1 Institute for Interdisciplinary Information Sciences, Tsinghua University, 100084 Beijing, China; 2 CAS Key Laboratory of Genomic and Precision Medicine, Beijing Institute of Genomics, Chinese Academy of Sciences and China National Center for Bioinformation, 100101 Beijing, China; 3 School of Computer Science and Technology, Xidian University, 710071 Xi’an, Shaanxi Province China; 4 Present Address: School of Engineering, Westlake University, 310030 Hangzhou, Zhejiang Province China

**Keywords:** Computational biology and bioinformatics, Regulatory networks, Differentiation, Control theory, Biotechnology

Correction to: *Nature Communications* 10.1038/s41467-023-44103-3, published online 20 December 2023

The original version of this Article omitted a reference to previous work in Fiedler et al., 2013. This has been added as reference (Ref.77) at Methods: ‘The first network control-based method for driver gene identification is based on the feedback vertex set (FVS)^33,77^ with nonlinearities:’ and ‘According to the FVS-based method proposed by Mochizuki et al.^33^ and Fiedler et al.^77^, controlling all the nodes in the FVS is sufficient to drive the system to any of its attractors (i.e., cell states). Here, we used the extended FVS-based method proposed by Zañudo et al.^32^, which control all the source nodes (i.e., the nodes with in-degree 0) and the nodes in the FVS.’ Also, the original version contained an error in Fig. 2c, where the two yellow nodes in the example network diagram should have been labelled as both MFVS and MDS driver genes, aligning with the algorithms. These has been corrected in both the PDF and HTML versions of the Article.

