# Supplementary material for: Deciphering driver regulators of cell fate decisions from single-cell transcriptomics data with CEFCON
Source: Nat Commun. 2023 Dec 20;14:8459. doi: 10.1038/s41467-023-44103-3 (PMC10733330; doi:10.1038/s41467-023-44103-3)
Supplement: Supplementary file 1 — Supplementary Information [file 41467_2023_44103_MOESM1_ESM.pdf]

Supplementary Information:

Deciphering driver regulators of cell fate decisions from single-cell transcriptomics data with CEFCON

Peizhuo Wang [1,4], Xiao Wen [2], Han Li [1], Peng Lang [1], Shuya Li [1,4], Yipin Lei [1], Hantao Shu [1], Lin Gao [3], Dan Zhao [1,*], and Jianyang Zeng [1,4,*]

1 Institute for Interdisciplinary Information Sciences, Tsinghua University, 100084, Beijing, China.
2 CAS Key Laboratory of Genomic and Precision Medicine, Beijing Institute of Genomics, Chinese Academy of Sciences and China National Center for Bioinformation, 100101, Beijing, China.
3 School of Computer Science and Technology, Xidian University, 710071, Xi'an, Shaanxi Province, China.
4 Present address: School of Engineering, Westlake University, 310030, Hangzhou, Zhejiang Province, China.
∗ All correspondence should be addressed to zhaodan2018@tsinghua.edu.cn, and zengjy@westlake.edu.cn.

This document contains:

- Supplementary Notes
- Supplementary Figures 1-24
- Supplementary Tables 1-4

A. Supplementary Notes

A.1. The attention scoring function

Graph attention operates on graph-structured data, through leveraging the masked self-attentional layers to compute the correlations between nodes and dynamically update the feature embeddings of nodes. In our CEFCON framework, we focused more on the attention coefficients, which were used to construct the gene regulatory networks (GRNs). We mainly used the cosine similarity attention [1] (denoted as att_{COS}) in this paper and also discussed two other frequently used attention scoring functions, i.e., the scaled dot-product from Transformer [2] (denoted as att_{SD}) and the additive attention from GAT [3] (denoted as att_{AD}) (Supplementary Figure 20), both of which were provided as additional options in our package. All the above attention scoring functions are defined as follows:

$$
\begin{aligned}
att_{COS}(\mathbf{W}_a\mathbf{h}_i, \mathbf{W}_b\mathbf{h}_j) &= D_j \cdot \left| \frac{(\mathbf{W}_a\mathbf{h}_i)^T(\mathbf{W}_b\mathbf{h}_j)}{\|\mathbf{W}_a\mathbf{h}_i\| \cdot \|\mathbf{W}_b\mathbf{h}_j\|} \right|, \\
att_{SD}(\mathbf{W}_a\mathbf{h}_i, \mathbf{W}_b\mathbf{h}_j) &= D_j \cdot \left| \frac{(\mathbf{W}_a\mathbf{h}_i)^T(\mathbf{W}_b\mathbf{h}_j)}{\sqrt{d}} \right|, \\
att_{AD}(\mathbf{W}_a\mathbf{h}_i, \mathbf{W}_b\mathbf{h}_j) &= D_j \cdot \mathrm{LeakyReLU}\left(\mathbf{a}^T \left[\mathbf{W}_a\mathbf{h}_i \| \mathbf{W}_b\mathbf{h}_j\right]\right),
\end{aligned}
$$

where $\mathbf{h}_i$ and $\mathbf{h}_j$ stand for the features of node v_i and v_j, respectively, $\mathbf{W}_a$ and $\mathbf{W}_b$ stand for their corresponding learnable weight matrics, $\mathbf{a}$ stands for a weight vector, d stands for the dimensionality of node feature embeddings, D_j stands for the encoding of the differential expression level of gene v_j, $\|\cdot\|$ stands for the Euclidean norm, $[\cdot\|\cdot]$ stands for the concatenation operation, and T stands for the matrix transpose. Note that we modified the original attention scoring functions of cosine and scaled-dot product by using their absolute values since we only consider the strength of regulation.

A.2. Brief introduction of the baseline methods

A.2.1. GRN construction:

1) SCINET [4]. SCINET constructs the cell-type-specific interactomes through filtering a global reference network according to the input single-cell gene expression profiles. In particular, it introduces a statistical framework to compute cell-level gene interaction probabilities

and group-level gene interaction strengths.
2) GRNBoost2 [5]. GRNBoost2 selects the most important regulators for each gene based on a gradient boosting regression approach. It is a fast alternative to GENIE3 [6], which is originally developed for bulk RNA-seq data and also has an outstanding performance on single-cell RNA-seq data. Both these two methods are ensembled in the SCENIC framework [7]. In this paper, we only used GRNBoost2 for comparison due to its good balance between efficiency and accuracy.
3) DeepSEM [8]. DeepSEM is a deep generative model previously developed in our group that uses the beta variational autoencoder to generate the structural equation model (SEM) to infer the regulatory relationships between genes.
4) NetREX [9]. NetREX infers context-specific GRNs from a prior interaction network and gene expression profiles based on a network component analysis (NCA) model. It estimates TF activities and reconstructs the prior interaction network simultaneously such that the network structure and the predicted TF activities best explain the context-specific expression data.
5) CellOracle [10]. CellOracle uses a regularized linear machine-learning model to infer cell-cluster-specific GRNs from single-cell multi-omics data and then performs *in silico* TF perturbation. In our comparison, we treat the entire cell lineage as a cluster to obtain the corresponding GRN for comparison.
6) Random_NicheNet. This method refers to a baseline that randomly selects edges from the prior gene interaction network [11]. The number of selected edges is the same as in the ground-truth network.

CellOracle, NetREX and SCINET are prior-based methods, each of which requires a background network. We used the default parameters for all baseline methods.

A.2.2. Driver regulator identification:

1) VIPER [12]. VIPER is a network-based method for measuring protein activities through regulon enrichment analysis. It takes an appropriate cell context-specific regulatory network and the gene expression profiles of two states as inputs. In this paper, we selected the gene expression data of the start and end states during the trajectory as inputs to VIPER for a fair comparison.
2) ANANSE [13]: ANANSE predicts the key transcription factors (TFs) in cell fate determination based on the enhancer-related epigenomic data. In particular, it uses either ATAC-seq and/or H3K27ac ChIP-seq data besides gene expression data as inputs and pro-

poses an influence score based on the differential network and differential expression for key TF prediction. In this paper, we only used the ANANSE influence scores on the same GRNs as constructed by CEFCON for a fair comparison.

3) SCENIC [7]: SCENIC uses a database of TF binding motifs to filter the regulatory interactions inferred by GENIE3 [6] or GRNBoost2 [5]. It includes only those interactions in which the motifs of the TFs are enriched in the target gene's promoter regions (i.e. regulons) and then measures the TF activity based on the identified regulons. In our comparison, we used pySCENIC [14], a lightning-fast Python implementation of SCENIC, and used the GRNBoost2 method for GRN construction (which was also utilized as a baseline for the evaluation of GRN construction).

4) CellOracle [10]: CellOracle uses its inferred cell-type-specific GRN to find important TFs and then performs *in silico* TF perturbation. It provides multiple node importance metrics for gene ranking. In our comparison, we used the default degree centrality to measure the gene importance from its own inferred GRNs.

5) CellRouter [15]: CellRouter first obtains cell subpopulations and developmental trajectories by constructing a GRN, and then uses a GRN score to rank the transcriptional regulators, which is defined based on the correlation of the regulators with the trajectory progression, the correlation of their predicted targets, and the degree to which these target genes are regulated during a specific trajectory.

A.3. Brief introduction of the different prior gene interaction networks

1) NicheNet [11]: NicheNet provides a collection of ligand-receptor, intracellular signaling, and gene regulatory interactions from over 50 public data sources of mouse and human. In this study, the ligand-receptor interactions between cells were removed because we only focused on the gene interactions within individual cells. The data were downloaded from the Github webpage of NicheNet (https://github.com/saeyslab/nichenetr/tree/master/data).

2) Harmonizome [16]: Harmonizome is a collection of processed datasets from over 70 major online resources. The network data we used were downloaded via the OmnipathR toolkit [17], utilizing the 'nichenet_gr_network_harmonizome' and 'nichenet_signaling_network_harmonizome' functions.

3) InWeb_InBioMap [18]: InWeb_InBioMap is a scored human protein–protein interaction network with over 500,000 interactions. The network data were downloaded using 'inbiomap_download' function from the OmnipathR package [17].

4) PathwayCommons [19]: PathwayCommons is an integrated resource that provides information about biological pathways, including biochemical reactions, assembly of biomolecular complexes, transport and catalysis events, and physical interactions involving proteins, DNA, RNA, and small molecules. We obtained the latest version (v12) of the complete network directly from https://www.pathwaycommons.org/.

5) Omnipath (interaction) [17]: Omnipath is an integrated resource of literature-curated human signaling pathway information, which combines data from over 100 resources. It provides a web server as well as R and Python packages for data retrieval and downloading. In this study, we downloaded the data with the curated interactions via the OmnipathR toolkit [17], utilizing the 'import_omnipath_interactions' function.

B. Supplementary Figures

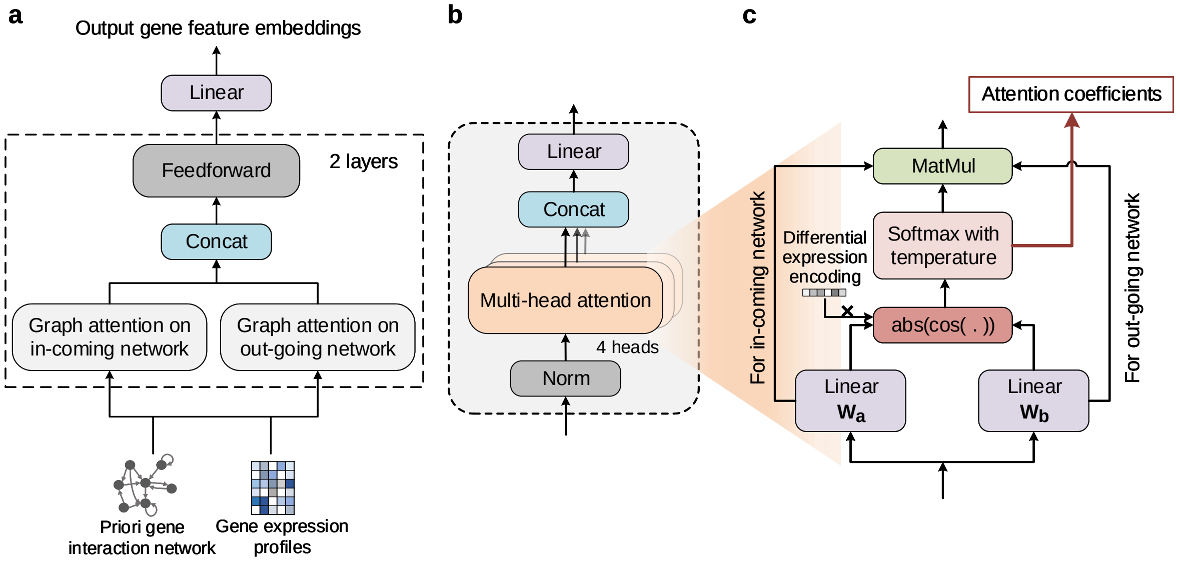

Supplementary Figure 1. Supplementary details on the GRN construction module in the CEFCON framework. a, The architecture of the encoder for GRN construction. **b,** The module of the employed graph attention neural network. **c,** The details of the attention mechanism. Abbreviations in the figure: Linear, linear layer; Feedforward, feedforward neural network; Concat, concatenation; Norm, batch normalization; abs, absolute; cos, cosine similarity; MatMul, matrix multiplication.

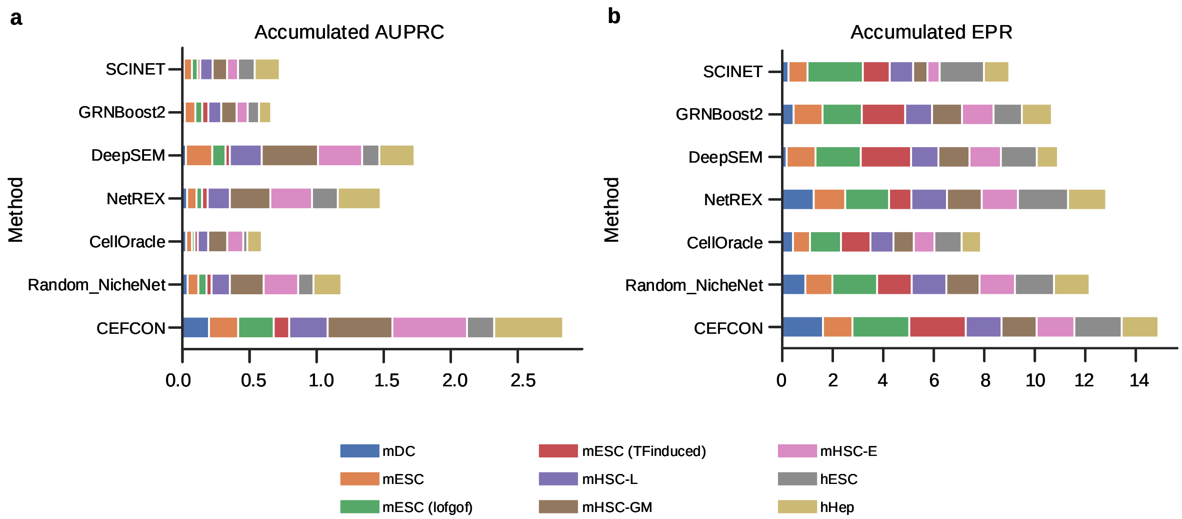

Supplementary Figure 2. The accumulated accuracy over all the benchmarking datasets on GRN construction. a, The accumulated AUPRC scores. **b,** The accumulated EPR scores. These results are related to Fig. 3 in the main text.

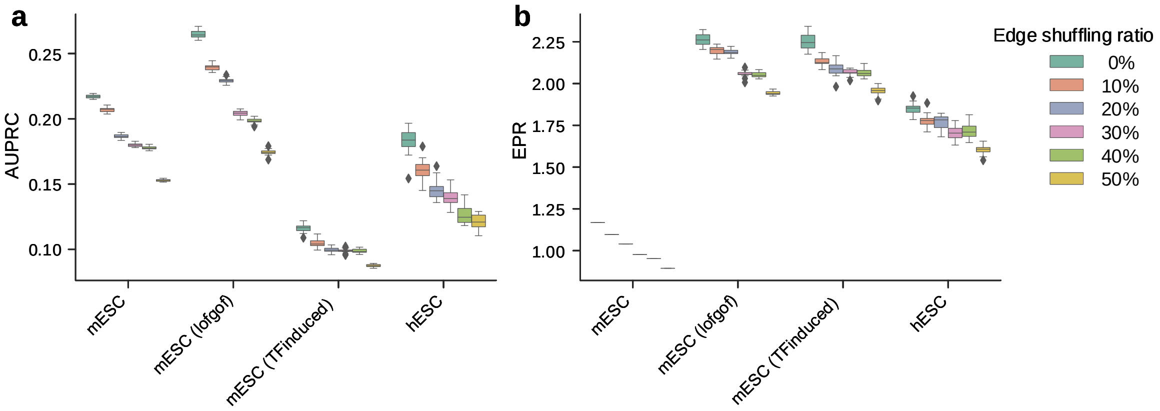

Supplementary Figure 3. The effect of edge perturbation of the prior gene interaction network on GRN construction, measured in terms of AUPRC (a) and EPR (b) on three benchmark datasets of mESC and a benchmark dataset of hESC. The accuracies on the original prior gene interaction network and the ones with five different shuffling ratios are reported. Each box-plot indicates the median (central line) and interquartile range, and the whisker represents 1.5×interquartile range, over n=20 independent computational experiments. Source data are provided as a Source Data file.

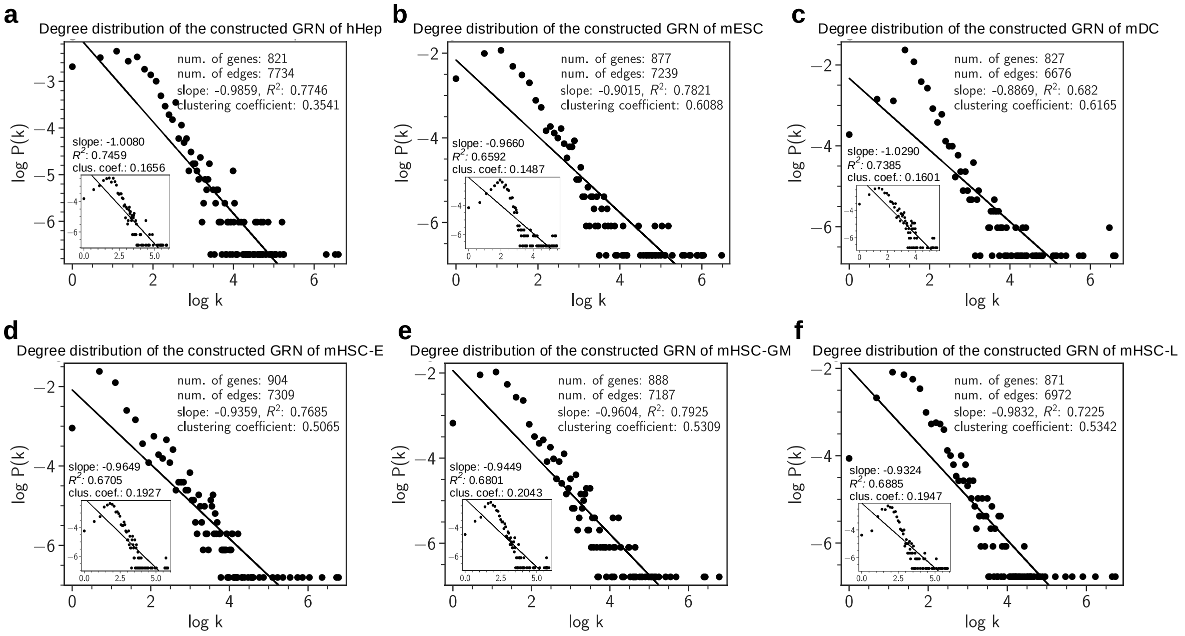

Supplementary Figure 4. Degree distributions and major topological properties of the GRNs constructed by CEFCON on the datasets of hHep (a), mESC (b), mDC (c), mHSC-E (d), mHSC-GM (e) and mHSC-L (f), respectively. The x-axis represents the network degree (denoted as k) and y-axis represents the frequency of the network degree k (denoted as $P(k)$). Both k and $P(k)$ are log-transformed. The R^2 is the coefficient of determination for the linear regression model to measure how close the data points are with respect to the fitted linear line. The major network topological properties, including the number of genes, the number of edges, the slope and the R^2 of the degree distribution, and the average clustering coefficient, were listed in the upper right corner of each figure. The inset in the bottom left corner of each figure provides the degree distributions (also with the slope, R^2 and average clustering coefficient) of the randomized GRN derived from the corresponding prior gene interaction network. Source data are provided as a Source Data file.

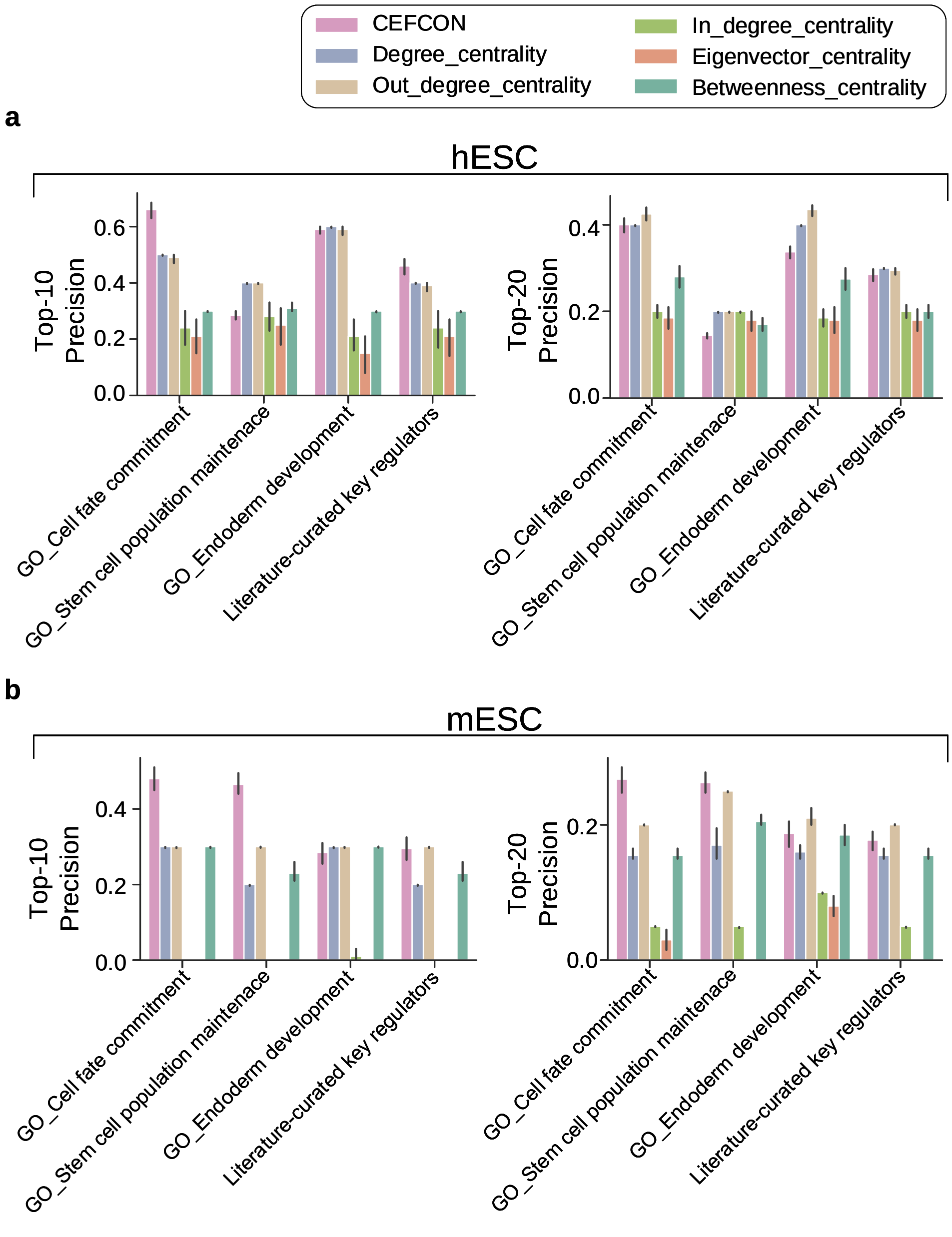

Supplementary Figure 5. Performance comparison of different node importance metrics on driver regulator identification based on the CEFCON constructed GRNs. a, The performance comparison on the hESC dataset. **b,** The performance comparison on the mESC dataset. Five node centrality metrics, i.e., degree centrality, out-degree centrality, in-degree centrality, eigenvector centrality and betweenness centrality, were used as baselines for comparison. These importance metrics were all calculated based on the same GRN constructed by CEFCON for each dataset. The precision values of the top-10 and top-20 predicted genes among all known genes in the four ground-truth gene sets were calculated, respectively. Bars and error bars signify mean ± s.d. over n=20 independent computational experiments. Source data are provided as a Source Data file.

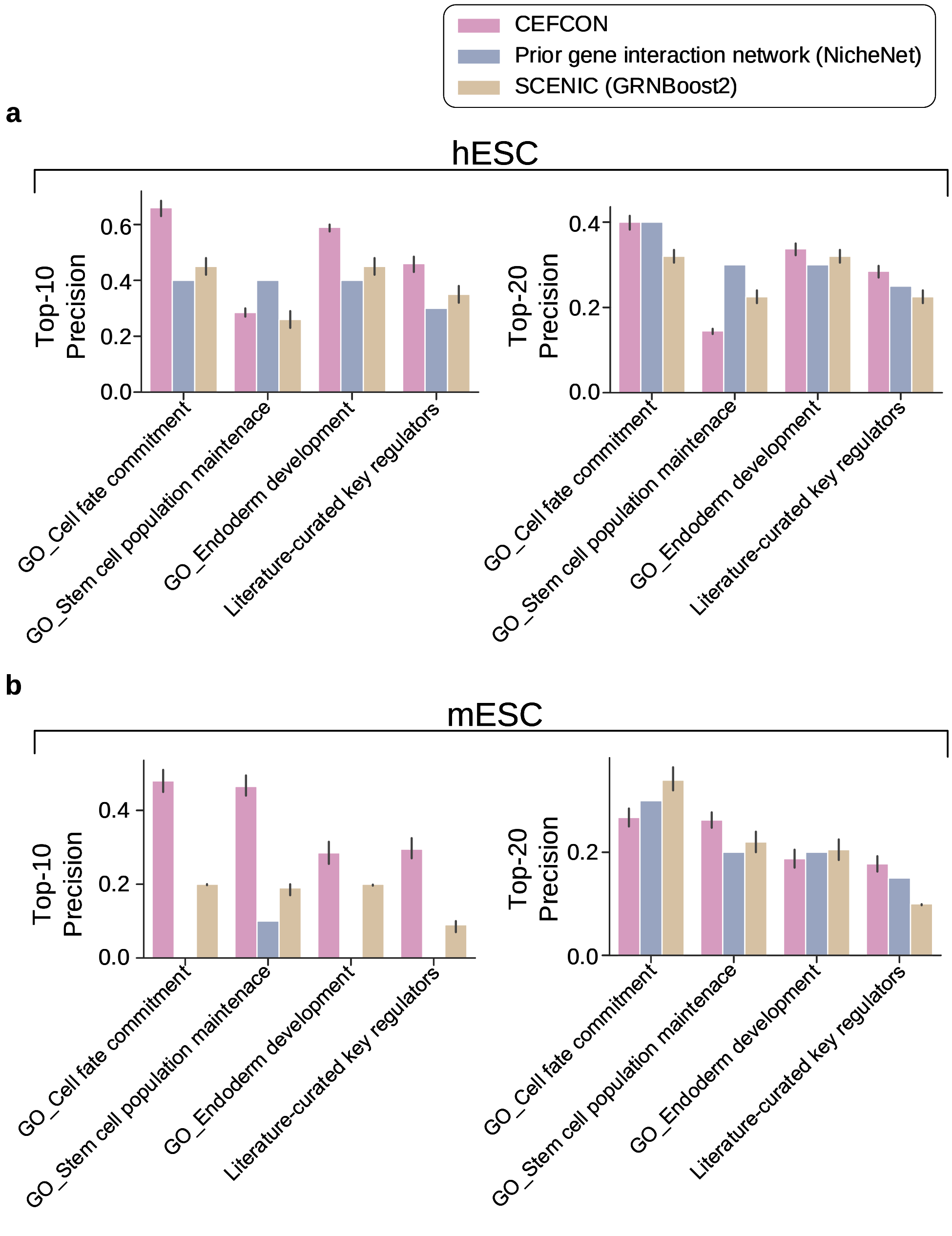

Supplementary Figure 6. Performance comparison on driver regulator identification using different constructed GRNs with the same network control methods (i.e., MDS and MFVS). a, The performance comparison on the hESC dataset. **b,** The performance comparison on the mESC dataset. The precision scores of the top-10 and top-20 predicted genes among all known genes in the four ground-truth gene sets were calculated, respectively. Regulators identified directly from the prior gene interaction network derived from NicheNet and those identified from the SCENIC-inferred GRNs were used for comparison with the results of CEFCON, respectively. Degree centrality was used for measuring the gene importance of the GRNs constructed by individual compared methods. Bars and error bars signify mean ± s.d. over n=20 independent computational experiments. Source data are provided as a Source Data file.

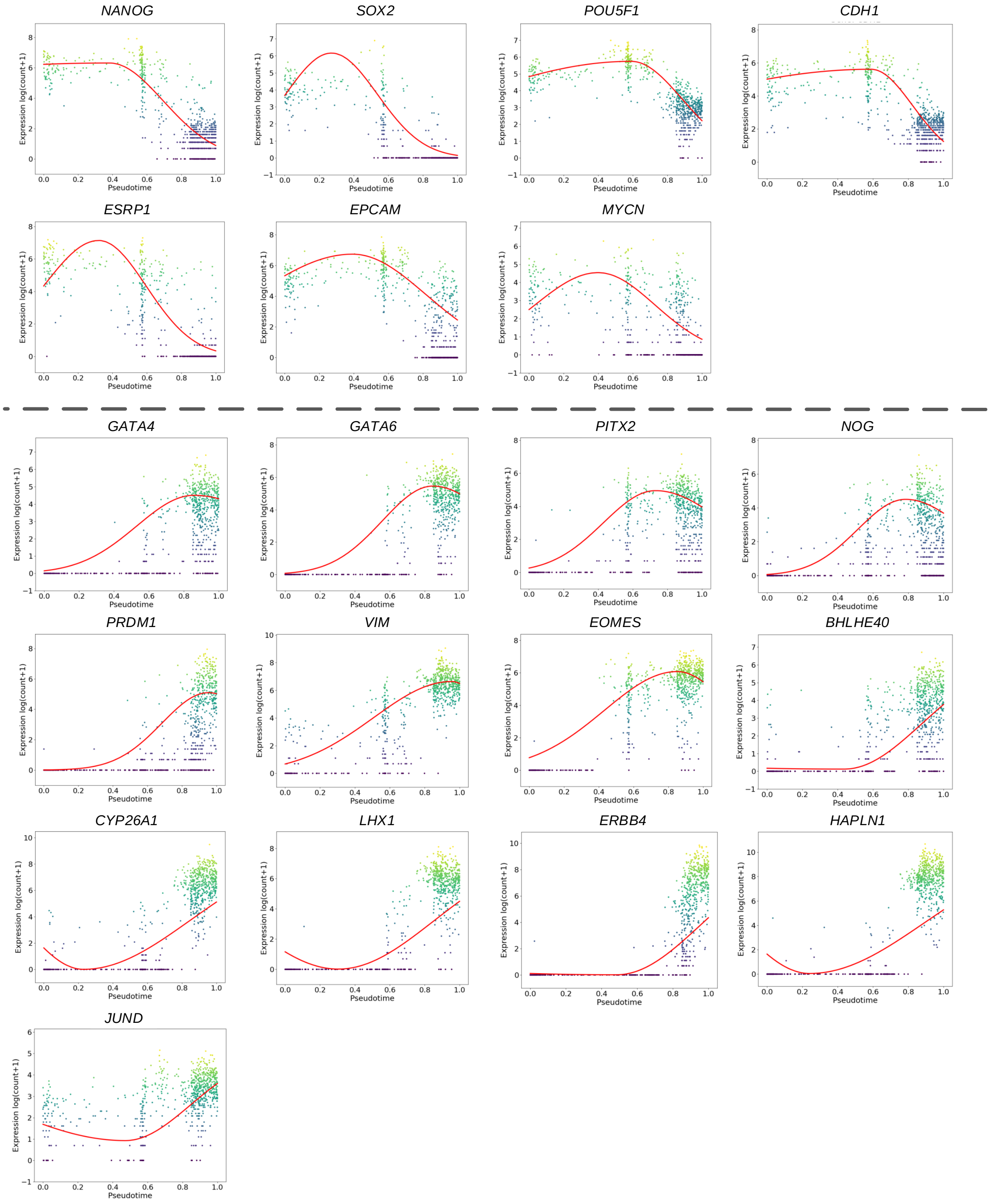

Supplementary Figure 7. The gene expression trends along the developmental pseudotime of the hESC dataset. Gene expression trends were obtained using the scGTM toolkit [20]. The top-20 genes identified by CEFCON are shown. These genes can be roughly divided into two groups based on their gene trends (separated by dotted lines), with a significant decrease or increase along the developmental pseudotime.

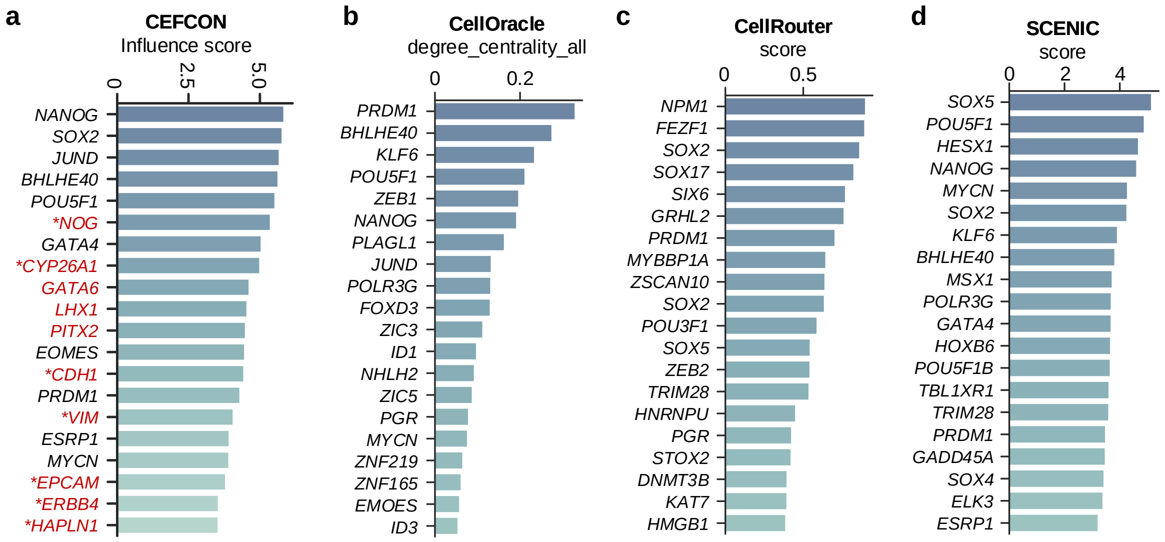

e

Gene	Description	References
NOG	Noggin (NOG) regulates embryonic development by inhibiting BMP (bone morphogenetic proteins) signaling.	[21], [22]
CYP26A1	CYP26A1 regulates retinoic acid levels, thereby affecting the differentiation process in endodermal cells.	[23], [24]
GATA6	GATA6 defines endoderm fate by controlling chromatin accessibility during differentiation of pluripotent stem cells.	[25], [26]
LHX1	LHX1 is involved in epiblast development in embryonic stem cells.	[27], [28]
PITX2	The PITX2 homeobox protein plays an essential role early in the nodal signaling pathway, directing the specification of both endodermal and mesodermal germ layers.	[29], [30]
CDH1	E-cadherin (CDH1) palys a vital role in maintaining cell–cell adhesions in embryonic stem cells (ESCs), which is crucial for embryonic stem cell pluripotency.	[31], [32]
VIM	Vimentin (VIM) is often used as a marker of mesenchymally derived cells or cells undergoing an epithelial-to-mesenchymal transition (EMT) during normal embryonic development.	[33]
EPCAM	EpCAM is a surface marker on undifferentiated hESCs and plays functional roles in proliferation and differentiation.	[34], [35]
ERBB4	ERBB4, which is a receptor tyrosine kinase, is linked to canonical and noncanonical Wnt signaling which can determine the cellular fate of human pluripotent stem cells.	[36], [37]
HAPLN1	HAPLN1 is a major component of the ECM (extracellular matrix), which is of great significance to tissue development (e.g., developmental hematopoiesis).	[38]

Supplementary Figure 8. The top-20 genes identified by different methods, including CEFCON (a), CellOracle (b), CellRouter (c), and SCENIC (d), on the hESC dataset. Genes uniquely detected by CEFCON are highlighted in red, and each non-TF is marked with an asterisk. **e,** Descriptions and related literature [21–38] of the genes uniquely identified by CEFCON among the top-20 predicted genes.

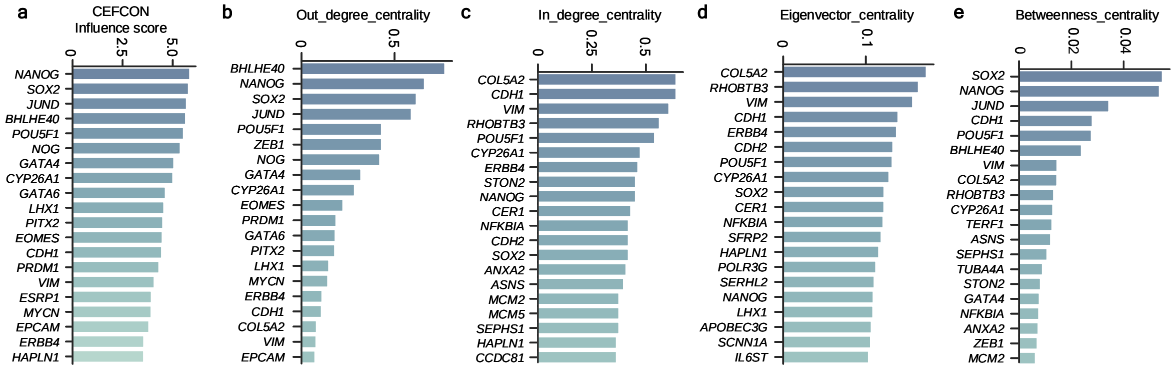

Supplementary Figure 9. The top-20 gene rankings of different node centrality measures, including the influence score of the CEFCON method (a), out-degree centrality (b), in-degree centrality (c), eigenvector centrality (d) and betweenness centrality (e), on the CEFCON constructed GRNs of the hESC dataset. The results showed that CEFCON identified both TFs and non-TFs more comprehensively than the other traditional centrality metrics.

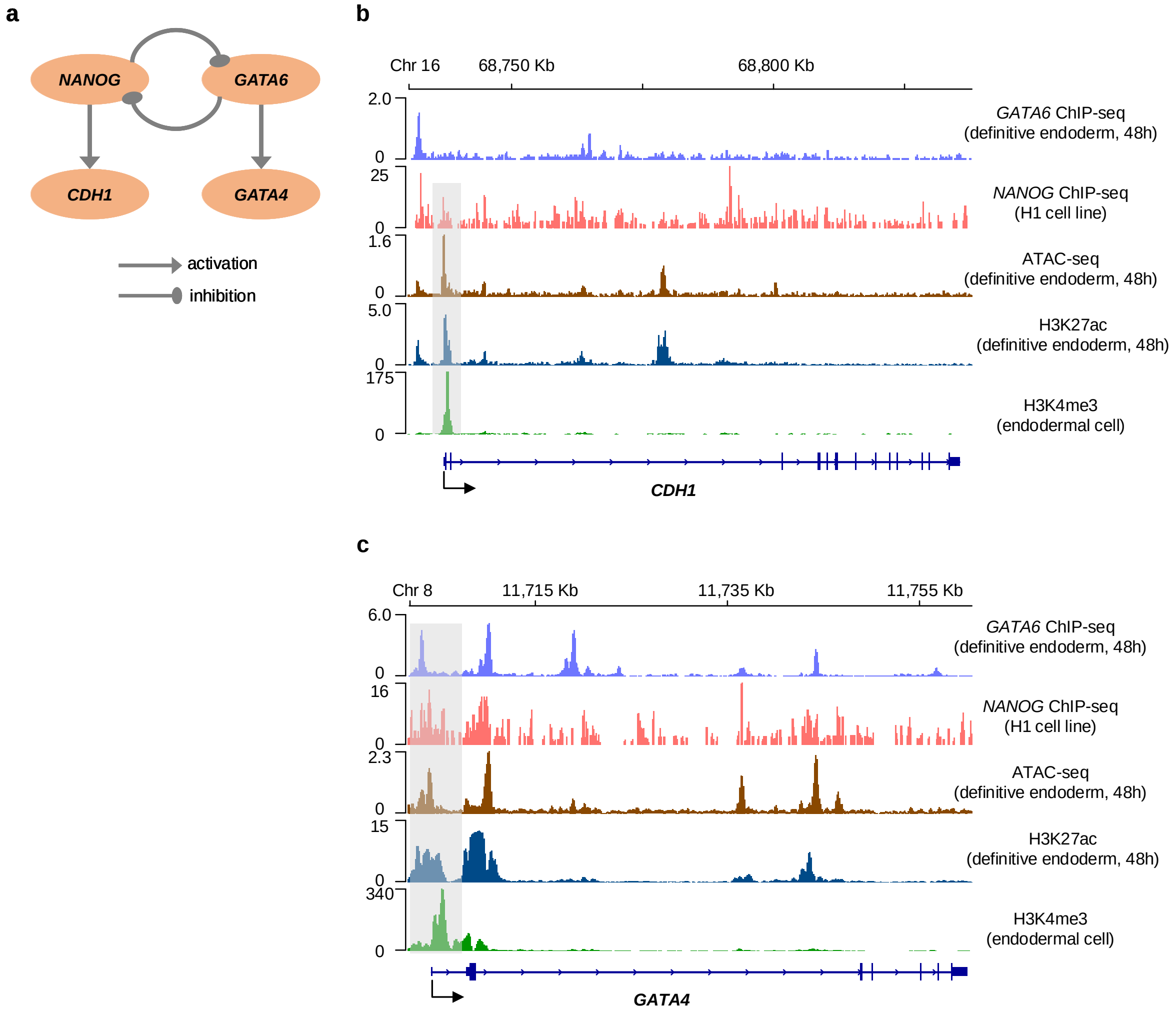

Supplementary Figure 10. Gene interactions between *NANOG*, *GATA6*, *CDH1* and *GATA4* predicted by CEFCON on the hESC dataset and their validation using the related ChIP-seq and epigenetic data. a, An illustration on the inferred interactions between *NANOG*, *GATA6*, *CDH1* and *GATA4*. **b-c,** The *GATA6* and *NANOG* ChIP-seq, ATAC-seq, H3K27ac and H3K4me3 peaks at promoters and gene bodies of *CDH1* **(b)** and *GATA4* **(c)** from the development of embryonic stem cells to definitive endoderm cells, visualized with the IGV genome browser [39]. The ATAC-seq, H3K27ac and *GATA6* ChIP-seq data at 48-hour post differentiation were from GEO (GSE213394). The H3K4me3 and *NANOG* ChIP-seq data were from the ENCODE database [40]. Shaded regions indicate the significant peaks in promoter regions of target genes.

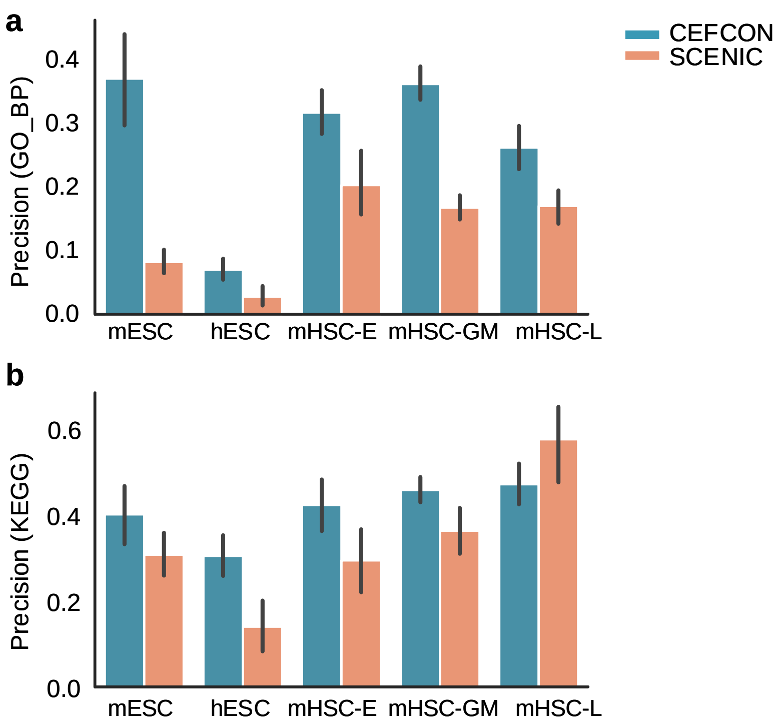

Supplementary Figure 11. Precision of the identified RGMs with at least 50 significantly enriched GO_BP terms (a) and at least five significantly enriched KEGG pathways (b) on the five tested datasets, respectively. Bars and error bars signify mean ± s.d. over n=20 independent computational experiments. Source data are provided as a Source Data file.

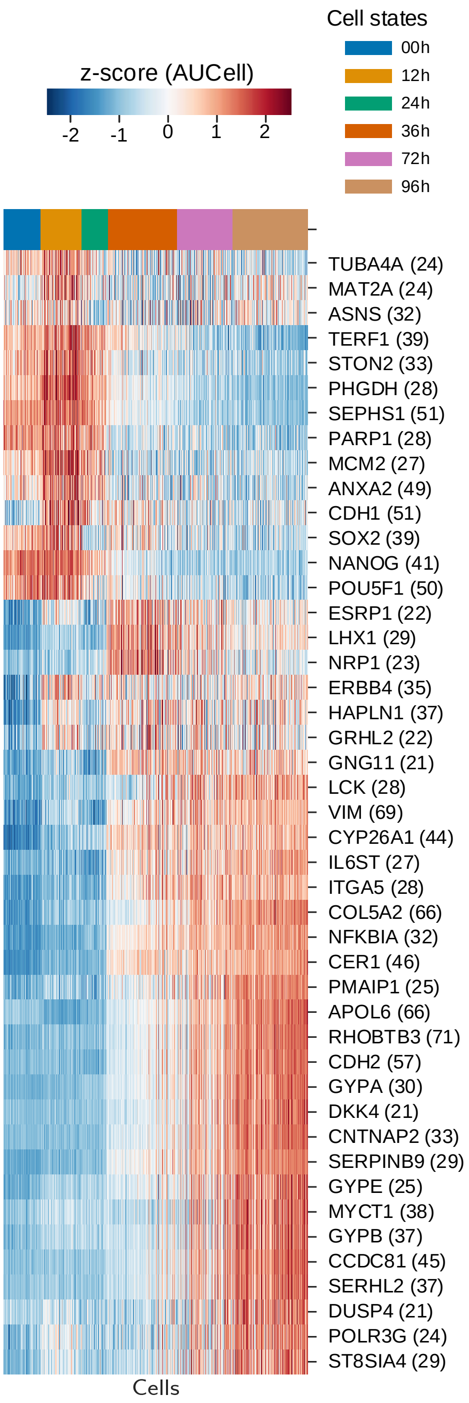

Supplementary Figure 12. The AUCell activity heatmap of the identified in-degree type of RGMs for the hESC dataset. Each RGM is represented by its involving driver regulator, and the number of member genes is given in parentheses. This result is related to Fig. 5e in the main text.

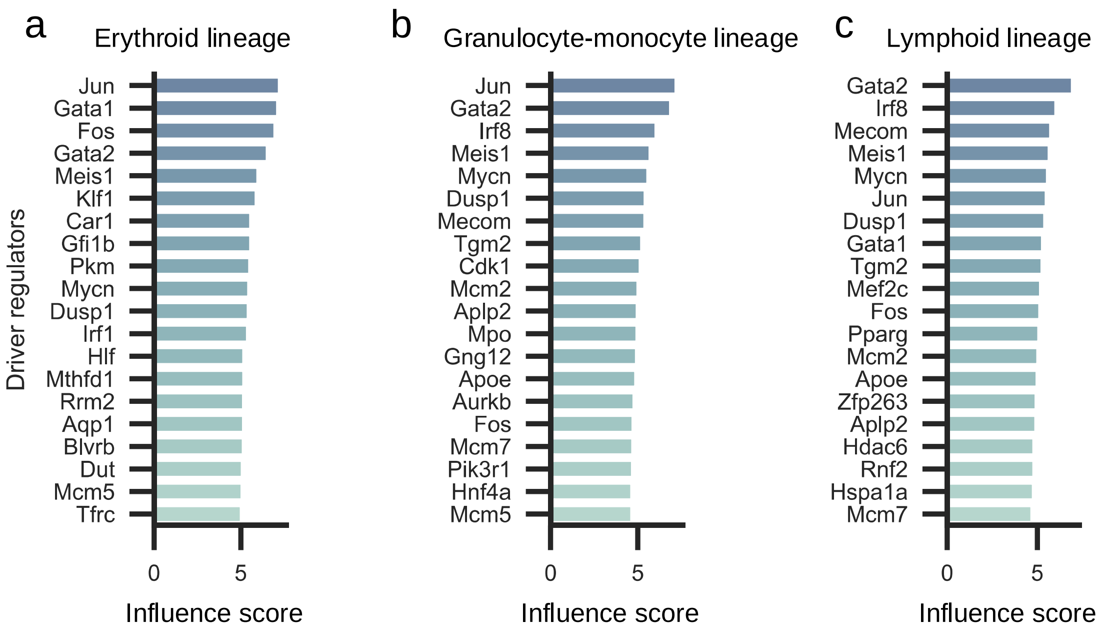

Supplementary Figure 13. The influence scores of the identified top-20 driver regulators for the three developmental lineages of mouse hematopoietic stem cell (mHSC) differentiation. a, Results on the erythroid lineage. **b,** Results on the granulocyte-monocyte lineage. **c,** Results on the lymphoid lineage. Source data are provided as a Source Data file.

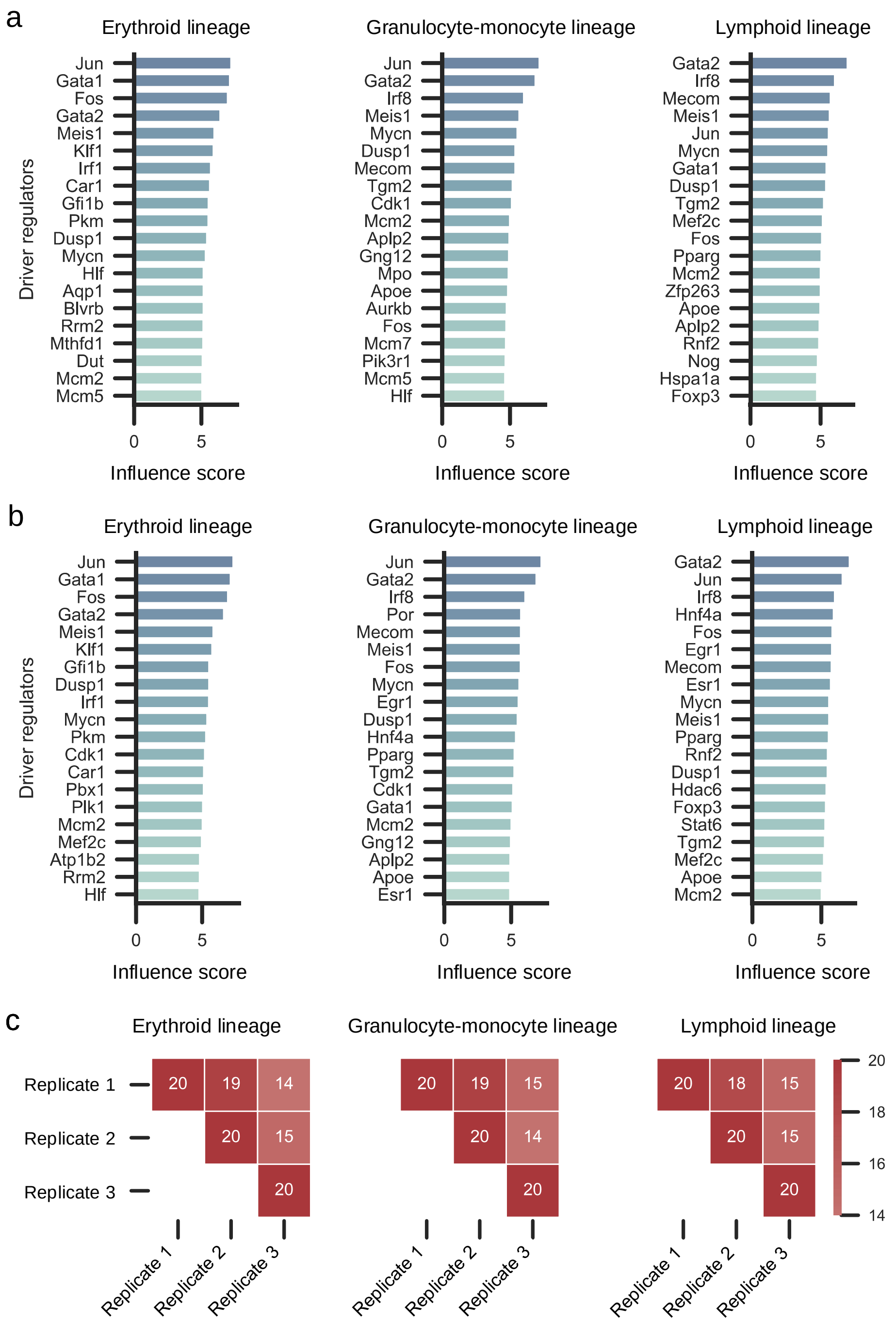

Supplementary Figure 14. Replicated results of the identified top-20 driver regulators for the three developmental lineages of mouse hematopoietic stem cell differentiation. These results were generated under different random seeds. The replicate 1 result was used for analyses in the main manuscript. **a,** The replicate 2 results. **b,** The replicate 3 results. **c,** The heatmaps illustrating the number of common genes between each pair of replicated results for the three developmental lineages. Source data are provided as a Source Data file.

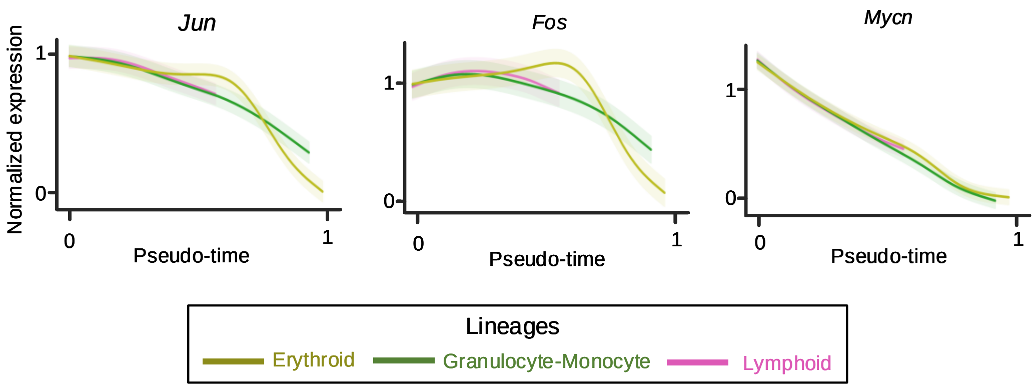

Supplementary Figure 15. The gene expression trends of the three identified common driver regulators identified by CEFCON, i.e., *Fos*, *Gata2*, *Mycn*, for the three developmental lineages of mHSC differentiation, as a supplement to Fig. 6d in the main text. The shaded area represents mean $\pm$ s.d..

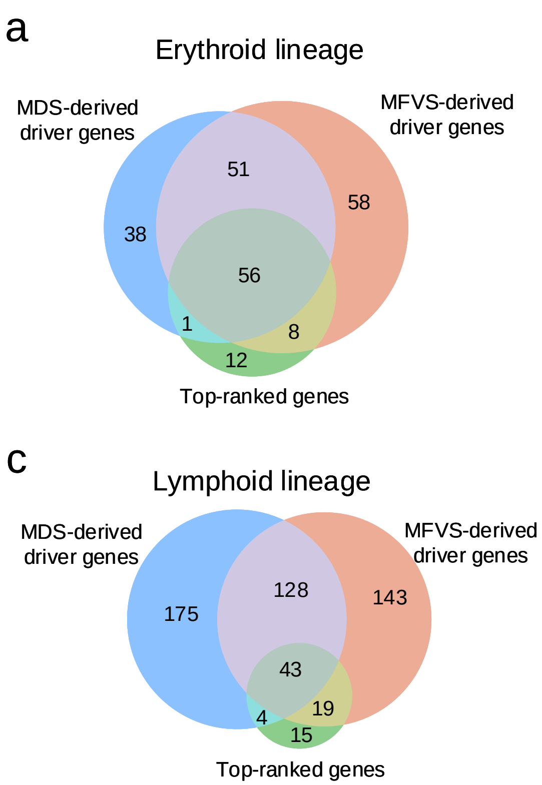

Supplementary Figure 16. Venn diagrams of the identified MDS-derived driver genes, MFVS-derived driver genes, and the top-ranked genes according to the influence scores for the three developmental lineages of mHSCs differentiation. **a,** Results on the erythroid lineage. **b,** Results on the granulocyte-monocyte lineage. **c,** Results on the lymphoid lineage.

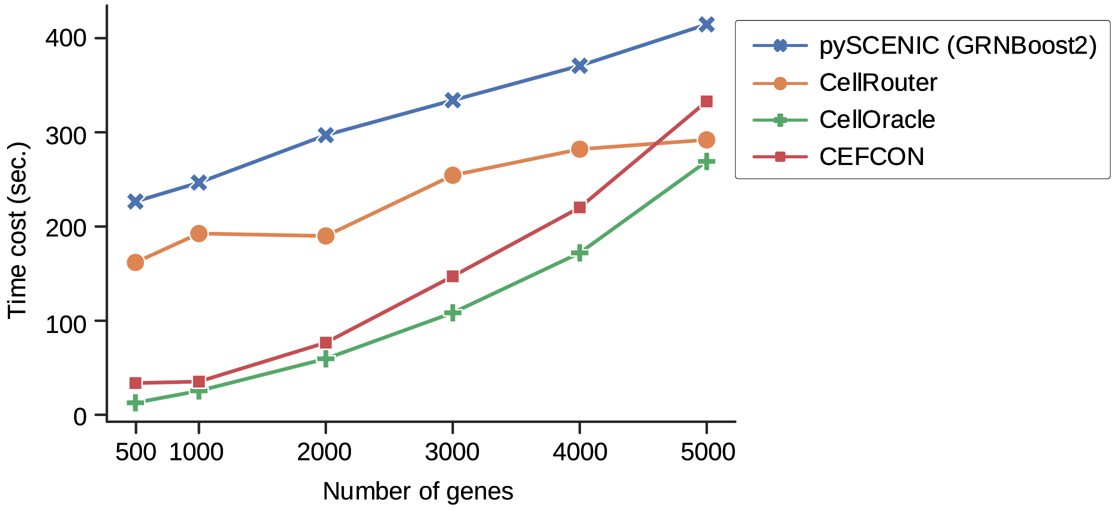

Supplementary Figure 17. The required time (in seconds) of different methods on the hESC dataset. The number of genes varied from 500 to 5,000. The time cost was the sum of the running time of both GRN construction and key gene identification. The tests were conducted on a 32-processor Linux server equipped with an AMD Ryzen Threadripper PRO 3975WX CPU (32 cores, 64 threads) and an NVIDIA A100 (40GB) GPU. We used the default parameters for all the compared methods. Source data are provided as a Source Data file.

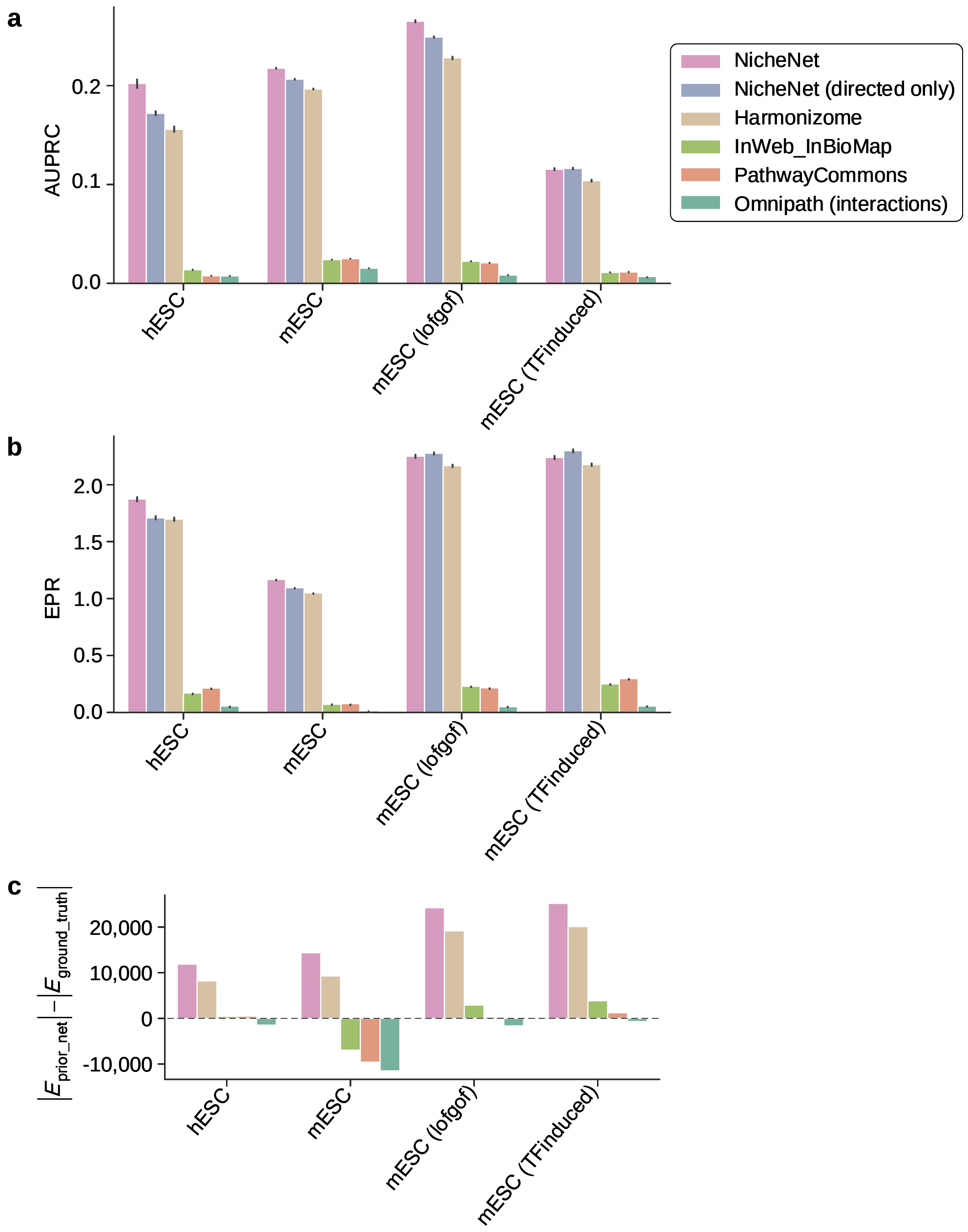

Supplementary Figure 18. Performance evaluation on GRN construction using different input prior gene interaction networks on the hESC and mESC datasets. **a,** The performance measured in terms of AUPRC. **b,** The performance measured in terms of EPR. The NicheNet network with only directed edges, i.e., NicheNet (directed only), and the other four baselines, including Harmonizome, InWeb_InBioMap, PathwayCommons, and Omnipath (interactions), were considered for comparison. Bars and error bars signify mean ± s.d. over n=20 independent computational experiments. **c,** The difference between the number of edges in the prior gene interaction networks (i.e., $|E_{prior\_net}|$) and the number of edges in the ground-truth network (i.e., $|E_{ground\_truth}|$). Source data are provided as a Source Data file.

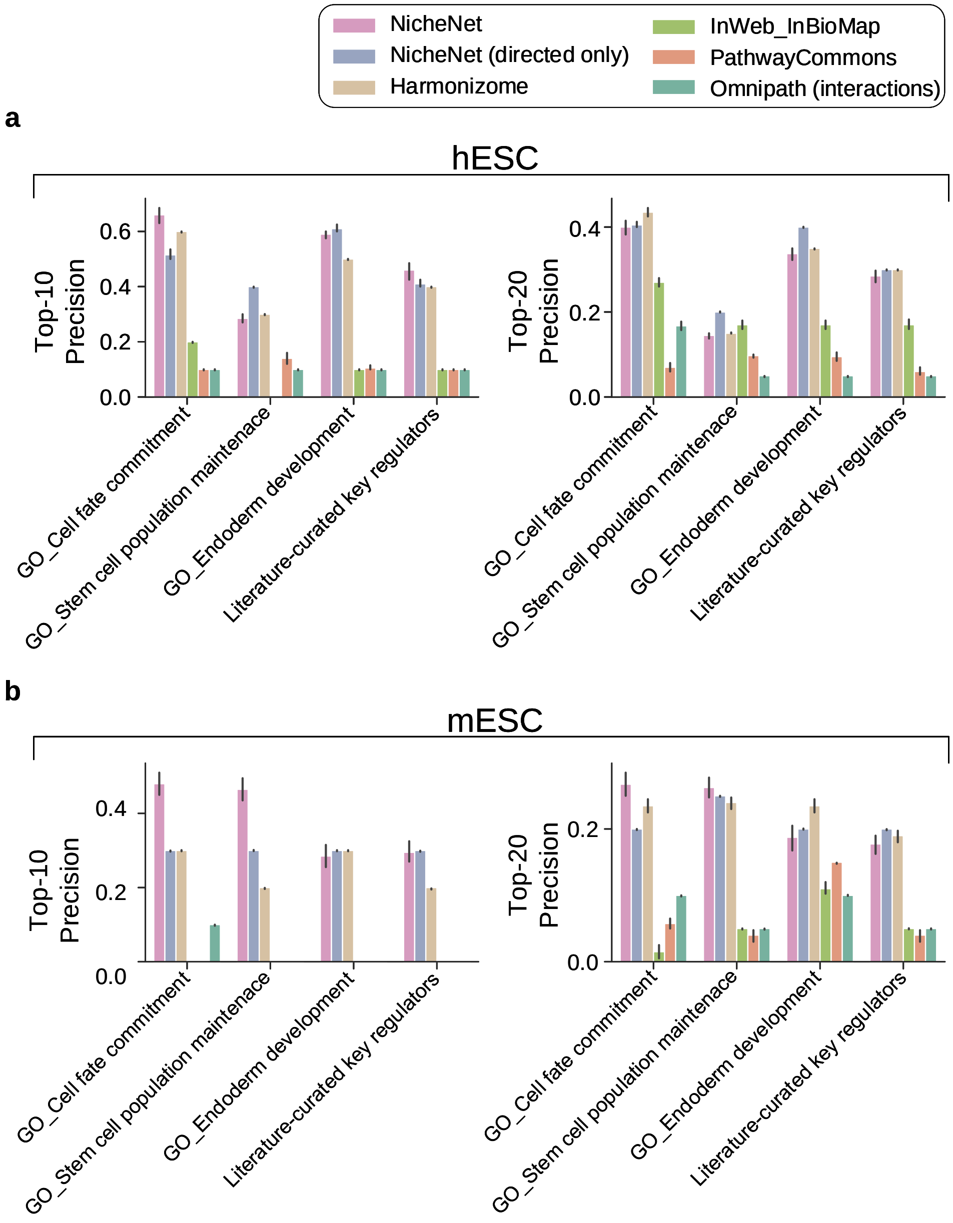

Supplementary Figure 19. Performance evaluation on driver regulator identification using different input prior gene interaction networks. a, The performance evaluation on the hESC dataset. **b,** The performance evaluation on the mESC dataset. The NicheNet network with only directed edges, i.e., NicheNet (directed only), and the other four baselines, including Harmonizome, InWeb_InBioMap, PathwayCommons, and Omnipath (interactions), were considered for comparison. The precision scores of the top-10 and top-20 predicted genes among all known genes in the four ground-truth gene sets were calculated, respectively. Bars and error bars signify mean ± s.d. over n=20 independent computational experiments. Source data are provided as a Source Data file.

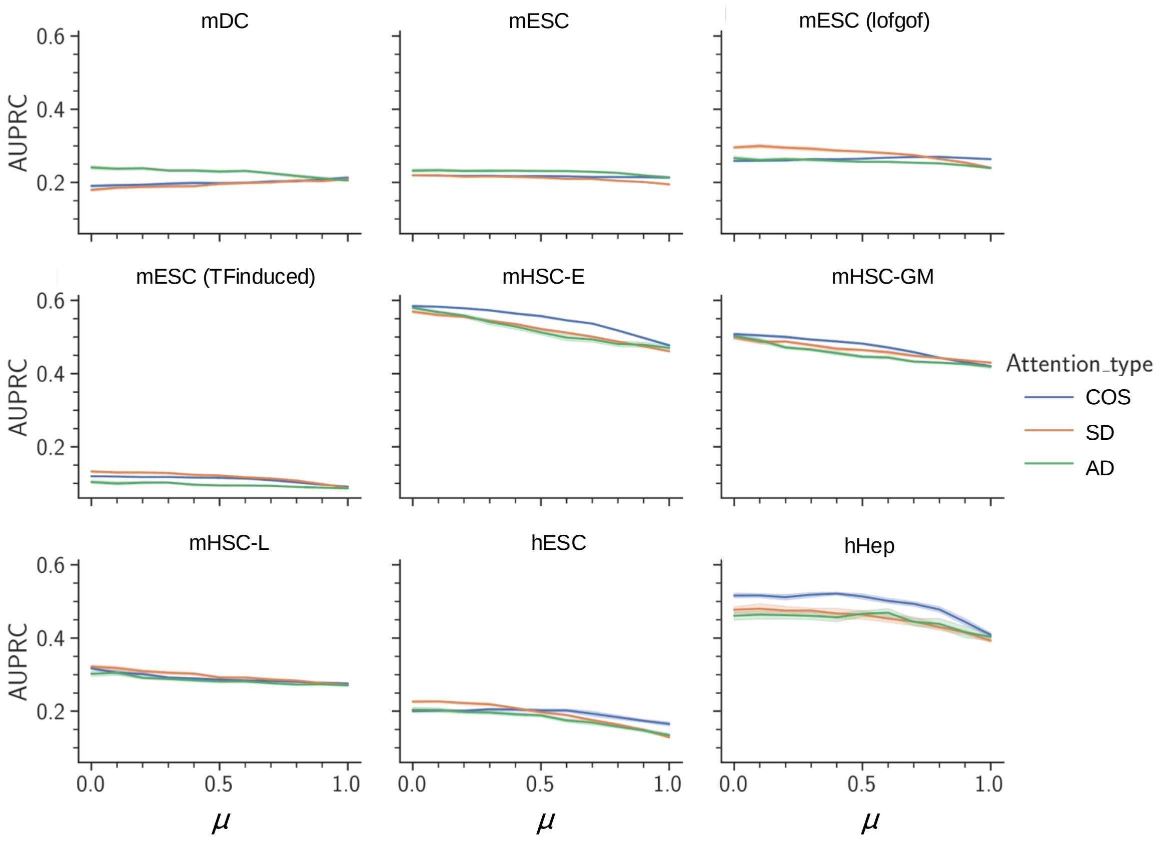

Supplementary Figure 20. AUPRC comparison of the three attention scoring functions on GRN construction with different values of parameter μ. The parameter μ balances the importance of attention coefficients between the first and second layers of GNN. The shaded area indicates the range of maximum and minimum values over 20 repeats. COS, the cosine similarity; SD, the scaled-dot product; AD, the additive attention. Source data are provided as a Source Data file.

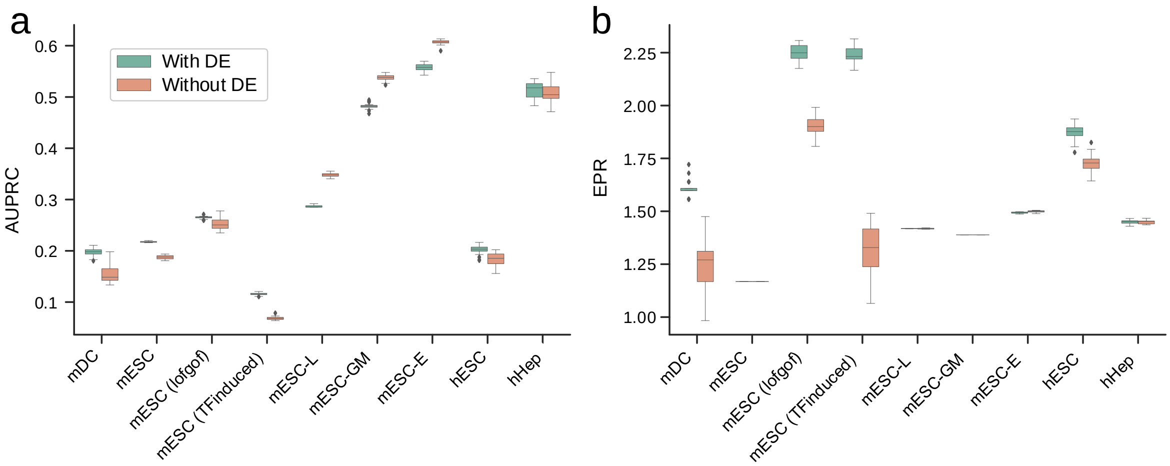

Supplementary Figure 21. The effect of encoding the gene differential expression on GRN construction on different benchmark datasets, measured in terms of AUPRC (a) and EPR (b). Each box-plot indicates the median (central line) and interquartile range, and the whisker represents 1.5×interquartile range, over n=20 independent computational experiments. DE, differential expression. Source data are provided as a Source Data file.

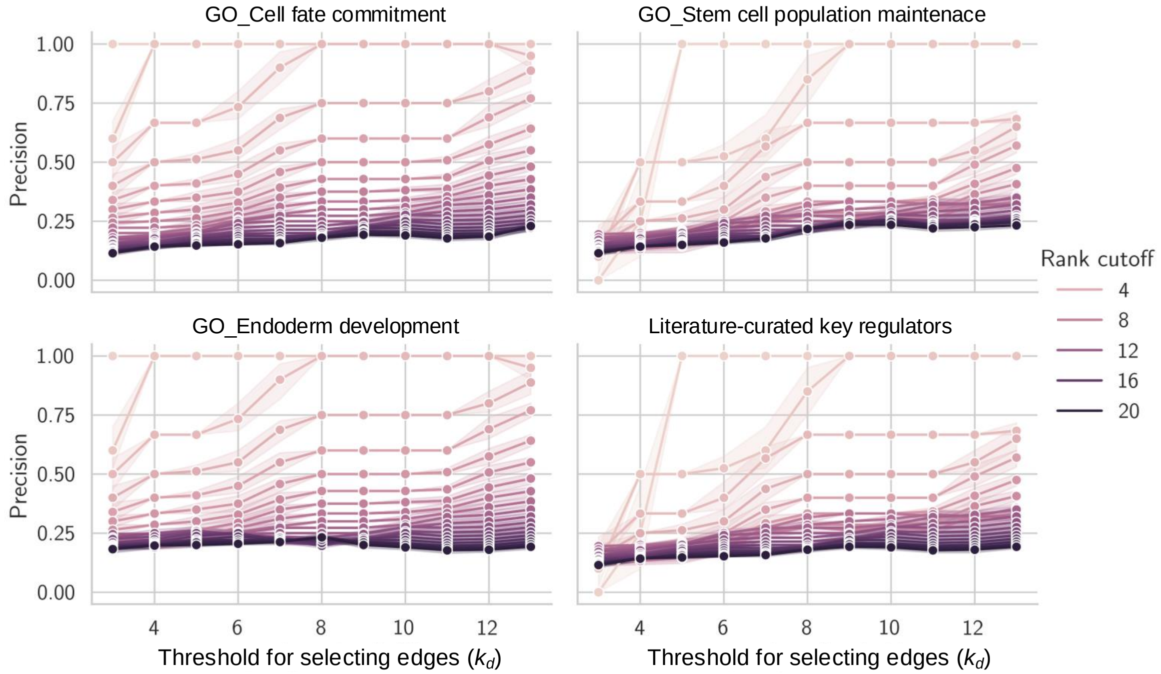

Supplementary Figure 22. The effect of different thresholds for selecting the top-weighted edges on the performance of driver regulator identification, measured in terms of the precision on the four ground-truth gene sets for the mESC dataset. The threshold parameter k_d (ranging from 3 to 13) is related to the average degree of the constructed GRNs. The results of the top-k (i.e., rank cutoff k from 1 to 20) driver regulators identified by CEFCON are given. The darker the color of the line, the higher the rank cutoff value. The shaded area indicates the range of maximum and minimum values over 20 repeats. Source data are provided as a Source Data file.

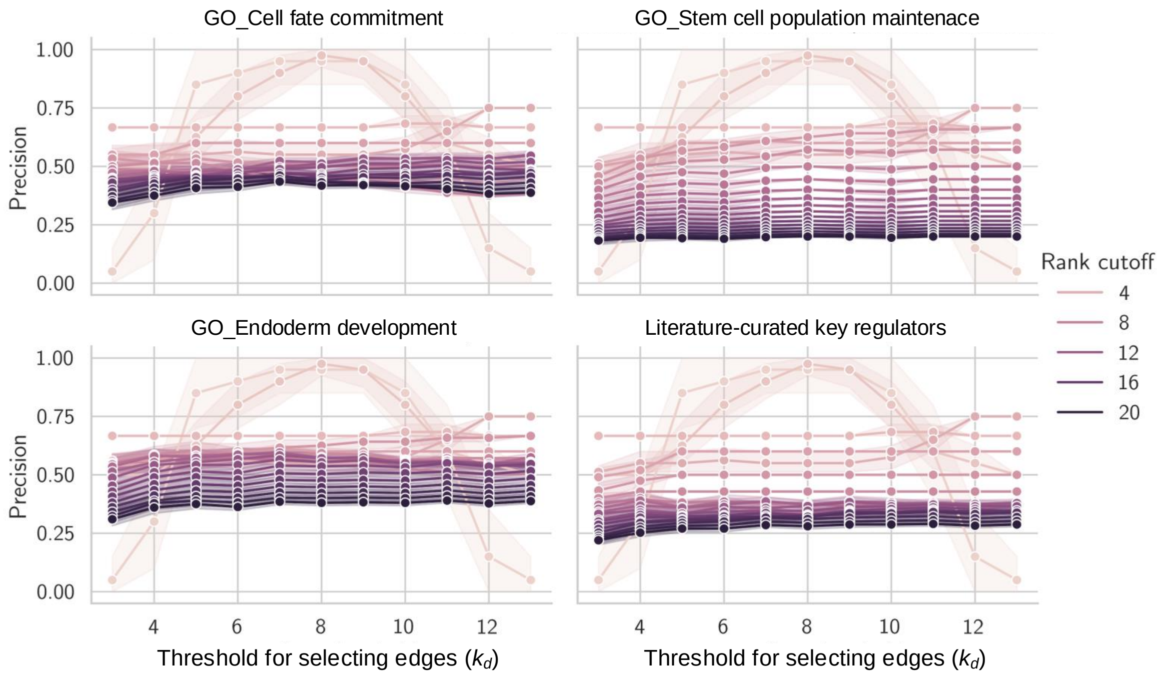

Supplementary Figure 23. The effect of different thresholds for selecting the top-weighted edges on the performance of driver regulator identification, measured in terms of the precision on the four ground-truth gene sets for the hESC dataset. The threshold parameter k_d (ranging from 3 to 13) is related to the average degree of the constructed GRNs. The results of the top-k (i.e., rank cutoff k from 1 to 20) driver regulators identified by CEFCON are given. The darker the color of the line, the higher the rank cutoff value. The shaded area indicates the range of maximum and minimum values over 20 repeats. Source data are provided as a Source Data file.

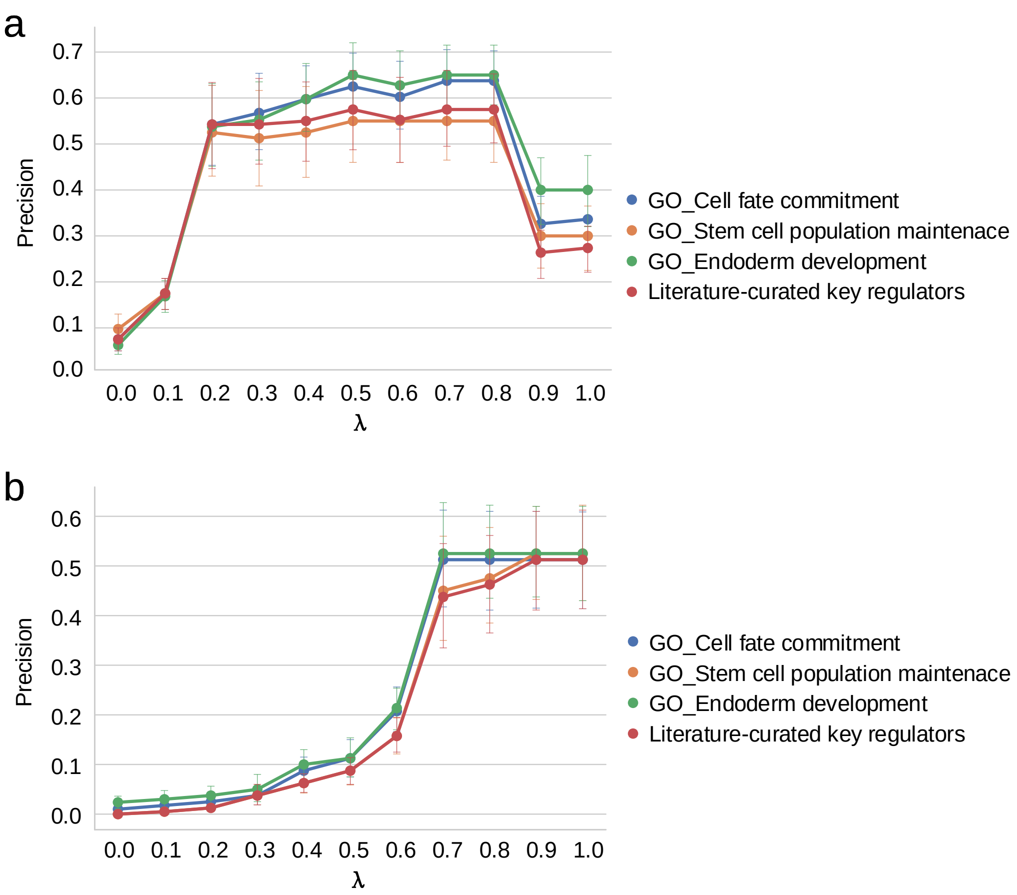

Supplementary Figure 24. The effect of different values of parameter λ on the performance of driver regulator identification, measured in terms of the precision on the four ground-truth gene sets. a, Results on the hESC dataset. **b,** Results on the mESC dataset. The parameter λ is used for balancing the importance of the influence scores between the in-coming and out-going networks. The results were averaged over all the top-k predictions, where k is taken from 1 to 20, and the whiskers represent the 1.5 $\times$ interquartile ranges. Source data are provided as a Source Data file.

C. Supplementary Tables

Supplementary Table 1. Statistics on the input prior gene interaction networks tested in this study.

Dataset	Species	#Source genes	#Target genes	#Total genes	#Edges	Avg. degree	Density	Avg. clustering coefficient	Slope of degree distribution (R^2)
NicheNet	Human	18,569	25,332	25,345	5,290,993	417.518	0.008	0.458	-0.953 (0.768)
	Mouse	17,455	18,579	18,579	5,029,532	541.421	0.015	0.420	-0.844 (0.678)
PathwayCommons	Human	16,688	18,689	19,087	1,105,240	115.811	0.003	0.138	-1.324 (0.864)
	Mouse	15,832	17,590	17,834	1,098,689	123.213	0.003	0.137	-1.299 (0.845)
InBioMap	Human	14,462	16,075	17,430	609,015	69.881	0.002	0.147	-1.347(0.877)
	Mouse	13,742	15,350	16,438	554,357	67.448	0.002	0.149	-1.324 (0.864)
Harmonizome	Human	4,319	26,381	26,780	2,985,645	222.976	0.004	0.492	-0.864 (0.607)
	Mouse	3,836	18,002	18,002	2,446,888	271.846	0.008	0.567	-0.751 (0.529)
Omnipath (interaction)	Human	6,182	7,435	8,725	86,248	19.770	0.001	0.197	-1.386 (0.796)
	Mouse	6,154	7,524	8,726	79,693	18.266	0.001	0.197	-1.406 (0.824)

Note: R^2 is the coefficient of determination for the linear regression model to measure how close the data points are with respect to the fitted linear line.

Supplementary Table 2. Statistics on the prior gene interaction networks with the top 1000 highly-variable genes for each scRNA-seq dataset used in this study.

Dataset	#Source genes	#Target genes	#Edges	Density (all)	Density (TFs)
mESC	863	920	47,863	0.053	0.169
mHSC-E	865	931	46,058	0.053	0.205
mHSC-GM	835	906	38,361	0.047	0.186
mHSC-L	762	874	22,435	0.029	0.167
mDC	857	865	57,201	0.077	0.148
hESC	761	924	20,792	0.024	0.08
hHEP	905	968	44,7099	0.048	0.135

Note: The density (all) is defined as the ratio of the actual number of edges to the maximum number of possible edges in the network, while the density (TFs) is the density of the sub-network considering only the edges outgoing from TFs.

Supplementary Table 3. Statistics on the ground-truth networks used to evaluate the constructed GRNs.

Dataset	#Data source	#TFs	#Genes	#Edges	Density
	ChIP-Atlas [41]/ESCAPE [42]	247	25,703	6,348,394	0.154
mESC	LOF/GOF [42]	57	18,427	104,797	0.1
	TFinduced [43]	134	15,184	140,157	0.069
mHSC	ChIP-Atlas [41]	137	19,324	1,078,888	0.407
mDC	ChIP-Atlas [41]	36	11,092	30,658	0.077
hESC	ChIP-Atlas [41]	130	18,104	436,563	0.186
hHEP	ChIP-Atlas [41]	84	16,822	342,862	0.243

Note: The density is computed only considering the edges outgoing from TFs.

Supplementary Table 4. The in-degree and out-degree statistics of TFs and non-TFs of the GRN constructed by CEFCON for each scRNA-seq dataset used in this study. The top 1,000 highly-variable genes were considered for GRN construction.

Dataset	#TFs	#non-TFs	Avg. in-degree of TFs	Avg. out-degree of TFs	Avg. in-degree of non-TFs	Avg. out-degree of non-TFs
hESC	86	819	9.4070	52.7326	3.4652	8.0147
hHEP	49	772	6.3061	29.6939	8.1334	9.6179
mESC	104	773	10.2788	29.6346	5.3777	7.9819
mDC	51	776	11.5882	65.4510	4.3015	7.8415
mHSC-E	58	846	10.5000	71.2931	3.7518	7.9196
mHSC-GM	66	822	8.5606	43.6515	5.2384	8.0560
mHSC-L	74	797	8.5541	48.5000	4.2447	7.9536

Supplementary References

[1] Thekumparampil, K. K., Wang, C., Oh, S. & Li, L.-J. Attention-based graph neural network for semi-supervised learning. *arXiv preprint arXiv:1803.03735* (2018). URL https://arxiv.org/abs/1803.03735 .

[2] Vaswani, A. *et al.* Attention is all you need. *Advances in neural information processing systems* **30** (2017). URL https://proceedings.neurips.cc/paper/2017/hash/3f5ee243547dee91fbd053c1c4a845aa-Abstract.html .

[3] Veličković, P. *et al.* Graph attention networks. *International Conference on Learning Representations* (2018). URL https://openreview.net/forum?id=rJXMpikCZ .

[4] Mohammadi, S., Davila-Velderrain, J. & Kellis, M. Reconstruction of cell-type-specific interactomes at single-cell resolution. *Cell Systems* **9** (6), 559–568.e4 (2019). URL https://doi.org/10.1016/j.cels.2019.10.007 .

[5] Moerman, T. *et al.* Grnboost2 and arboreto: efficient and scalable inference of gene regulatory networks. *Bioinformatics* **35** (12), 2159–2161 (2019). URL https://doi.org/10.1093/bioinformatics/bty916 .

[6] Huynh-Thu, V. A., Irrthum, A., Wehenkel, L. & Geurts, P. Inferring regulatory networks from expression data using tree-based methods. *PLOS ONE* **5** (9), 1–10 (2010). URL https://doi.org/10.1371/journal.pone.0012776 .

[7] Aibar, S. *et al.* Scenic: single-cell regulatory network inference and clustering. *Nature Methods* **14** (11), 1083–1086 (2017). URL https://doi.org/10.1038/nmeth.4463 .

[8] Shu, H. *et al.* Modeling gene regulatory networks using neural network architectures. *Nature Computational Science* **1** (7), 491–501 (2021). URL https://doi.org/10.1038/s43588-021-00099-8 .

[9] Wang, Y. *et al.* Reprogramming of regulatory network using expression uncovers sex-specific gene regulation in drosophila. *Nature Communications* **9** (1), 4061 (2018). URL https://doi.org/10.1038/s41467-018-06382-z .

[10] Kamimoto, K. *et al.* Dissecting cell identity via network inference and in silico gene perturbation. *Nature* **614** (7949), 742–751 (2023). URL https://doi.org/10.1038/s41586-022-05688-9.

[11] Browaeys, R., Saelens, W. & Saeys, Y. Nichenet: modeling intercellular communication by linking ligands to target genes. *Nature Methods* **17** (2), 159–162 (2020). URL https://doi.org/10.1038/s41592-019-0667-5 .

[12] Alvarez, M. J. *et al.* Functional characterization of somatic mutations in cancer using network-based inference of protein activity. *Nature Genetics* **48** (8), 838–847 (2016). URL https://doi.org/10.1038/ng.3593 .

[13] Xu, Q. *et al.* Ananse: an enhancer network-based computational approach for predicting key transcription factors in cell fate determination. *Nucleic Acids Research* **49** (14), 7966–7985 (2021). URL https://doi.org/10.1093/nar/gkab598 .

[14] Van De Sande, B. *et al.* A scalable scenic workflow for single-cell gene regulatory network analysis. *Nature Protocols* **15** (7), 2247–2276 (2020). URL https://dx.doi.org/10.1038/s41596-020-0336-2 .

[15] Lummertz da Rocha, E. *et al.* Reconstruction of complex single-cell trajectories using cellrouter. *Nature Communications* **9** (1), 892 (2018). URL https://doi.org/10.1038/s41467-018-03214-y.

[16] Rouillard, A. D. *et al.* The harmonizome: a collection of processed datasets gathered to serve and mine knowledge about genes and proteins. *Database* **2016**, baw100 (2016). URL https://doi.org/10.1093/database/baw100.

[17] Türei, D., Korcsmáros, T. & Saez-Rodriguez, J. Omnipath: guidelines and gateway for literature-curated signaling pathway resources. *Nature Methods* **13** (12), 966–967 (2016). URL https://doi.org/10.1038/nmeth.4077.

[18] Li, T. *et al.* A scored human protein–protein interaction network to catalyze genomic interpretation. *Nature Methods* **14** (1), 61–64 (2017). URL https://doi.org/10.1038/nmeth.4083.

[19] Rodchenkov, I. *et al.* Pathway Commons 2019 Update: integration, analysis and exploration of pathway data. *Nucleic Acids Research* **48** (D1), D489–D497 (2019). URL https://doi.org/10.1093/nar/gkz946.

[20] Cui, E. H., Song, D., Wong, W. K. & Li, J. J. Single-cell generalized trend model (scGTM): a flexible and interpretable model of gene expression trend along cell pseudotime. *Bioinformatics* **38** (16), 3927–3934 (2022). URL https://doi.org/10.1093/bioinformatics/btac423.

[21] Fausett, S. R., Brunet, L. J. & Klingensmith, J. Bmp antagonism by noggin is required in presumptive notochord cells for mammalian foregut morphogenesis. *Developmental Biology* **391** (1), 111–124 (2014). URL https://www.sciencedirect.com/science/article/pii/S0012160614000761.

[22] Tropepe, V. *et al.* Direct neural fate specification from embryonic stem cells: a primitive mammalian neural stem cell stage acquired through a default mechanism. *Neuron* **30** (1), 65–78 (2001). URL https://doi.org/10.1016/S0896-6273(01)00263-X .

[23] Jin, L. *et al.* Serine threonine kinase receptor-associated protein deficiency impairs mouse embryonic stem cells lineage commitment through cyp26a1-mediated retinoic acid homeostasis. *Stem Cells* **36** (9), 1368–1379 (2018). URL https://doi.org/10.1002/stem.2854 .

[24] Russo, L., Sladitschek, H. L. & Neveu, P. A. Multi-layered regulation of neuroectoderm differentiation by retinoic acid in a primitive streak-like context. *Stem Cell Reports* **17** (2), 231–244 (2022). URL https://www.sciencedirect.com/science/article/pii/S2213671121006536.

[25] Fujikura, J. *et al.* Differentiation of embryonic stem cells is induced by gata factors. *Genes & development* **16** (7), 784–789 (2002). URL http://www.genesdev.org/cgi/doi/10.1101/gad.968802 .

[26] Thompson, J. J. *et al.* Extensive co-binding and rapid redistribution of nanog and gata6 during emergence of divergent lineages. *Nature Communications* **13** (1), 4257 (2022). URL https://doi.org/10.1038/s41467-022-31938-5.

[27] Costello, I. *et al.* Lhx1 functions together with otx2, foxa2, and ldb1 to govern anterior mesendoderm, node, and midline development. *Genes & development* **29** (20), 2108–2122 (2015) .

[28] McMahon, R., Sibbritt, T., Salehin, N., Osteil, P. & Tam, P. P. L. Mechanistic insights from the lhx1-driven molecular network in building the embryonic head. *Development, Growth & Differentiation* **61** (5), 327–336 (2019). URL https://onlinelibrary.wiley.com/doi/abs/10.1111/dgd.12609.

[29] Faucourt, M., Houliston, E., Besnardeau, L., Kimelman, D. & Lepage, T. The pitx2 homeobox protein is required early for endoderm formation and nodal signaling. *Developmental Biology* **229** (2), 287–306 (2001). URL https://www.sciencedirect.com/science/article/pii/S0012160600999500.

[30] Logan, M., Pagán-Westphal, S. M., Smith, D. M., Paganessi, L. & Tabin, C. J. The transcription factor pitx2 mediates situs-specific morphogenesis in response to left-right asymmetric signals. *Cell* **94** (3), 307–317 (1998). URL https://doi.org/10.1016/S0092-8674(00)81474-9 .

[31] Takeichi, M. Cadherin cell adhesion receptors as a morphogenetic regulator. *Science* **251** (5000), 1451–1455 (1991). URL https://doi.org/10.1126/science.2006419 .

[32] Redmer, T. *et al.* E-cadherin is crucial for embryonic stem cell pluripotency and can replace oct4 during somatic cell reprogramming. *EMBO reports* **12** (7), 720–726 (2011). URL https://www.embopress.org/doi/abs/10.1038/embor.2011.88.

[33] Mani, S. A. *et al.* The epithelial-mesenchymal transition generates cells with properties of stem cells. *Cell* **133** (4), 704–715 (2008). URL https://doi.org/10.1016/j.cell.2008.03.027 .

[34] González, B., Denzel, S., Mack, B., Conrad, M. & Gires, O. Epcam is involved in maintenance of the murine embryonic stem cell phenotype. *Stem Cells* **27** (8), 1782–1791 (2009). URL https://doi.org/10.1002/stem.97 .

[35] Ng, V. Y., Ang, S. N., Chan, J. X. & Choo, A. B. Characterization of epithelial cell adhesion molecule as a surface marker on undifferentiated human embryonic stem cells. *Stem Cells* **28** (1), 29–35 (2009). URL https://doi.org/10.1002/stem.221.

[36] Yarden, Y. & Sliwkowski, M. X. Untangling the erbb signalling network. *Nature Reviews Molecular Cell Biology* **2** (2), 127–137 (2001). URL https://doi.org/10.1038/35052073 .

[37] Ramachandra, C. J. *et al.* Erbb receptor tyrosine kinase: a molecular switch between cardiac and neuroectoderm specification in human pluripotent stem cells. *Stem cells* **34** (10), 2461–2470 (2016). URL https://doi.org/10.1002/stem.2420 .

[38] Mahony, C. B. *et al.* Hapln1b, a central organizer of the ECM, modulates kit signaling to control developmental hematopoiesis in zebrafish. *Blood Advances* **5** (23), 4935–4948 (2021). URL https://doi.org/10.1182/bloodadvances.2020001524.

[39] Robinson, J. T. *et al.* Integrative genomics viewer. *Nature Biotechnology* **29** (1), 24–26 (2011). URL https://doi.org/10.1038/nbt.1754.

[40] Dunham, I. *et al.* An integrated encyclopedia of dna elements in the human genome. *Nature* **489** (7414), 57–74 (2012). URL `https://doi.org/10.1038/nature11247`.

[41] Zou, Z., Ohta, T., Miura, F. & Oki, S. Chip-atlas 2021 update: a data-mining suite for exploring epigenomic landscapes by fully integrating chip-seq, atac-seq and bisulfite-seq data. *Nucleic Acids Research* **50** (W1), W175–W182 (2022). URL `https://doi.org/10.1093/nar/gkac199` .

[42] Xu, H. *et al.* Escape: database for integrating high-content published data collected from human and mouse embryonic stem cells. *Database* **2013** (2013), bat045 (2013). URL `https://doi.org/10.1093/database/bat045` .

[43] Correa-Cerro, L. S. *et al.* Generation of mouse es cell lines engineered for the forced induction of transcription factors. *Scientific Reports* **1** (1), 167 (2011). URL `https://doi.org/10.1038/srep00167` .
