# Supplementary material for: Deciphering driver regulators of cell fate decisions from single-cell transcriptomics data with CEFCON
Source: Nat Commun. 2023 Dec 20;14:8459. doi: 10.1038/s41467-023-44103-3 (PMC10733330; doi:10.1038/s41467-023-44103-3)
Supplement: Supplementary file 3 — Reporting Summary [file 41467_2023_44103_MOESM3_ESM.pdf]

nature portfolio

Corresponding author(s): Jianyang Zeng
Dan Zhao

Last updated by author(s): Nov 9, 2023

Reporting Summary

Nature Portfolio wishes to improve the reproducibility of the work that we publish. This form provides structure for consistency and transparency in reporting. For further information on Nature Portfolio policies, see our Editorial Policies and the Editorial Policy Checklist.

Statistics

For all statistical analyses, confirm that the following items are present in the figure legend, table legend, main text, or Methods section.

n/a	Confirmed	
☐	☒	The exact sample size (*n*) for each experimental group/condition, given as a discrete number and unit of measurement
☐	☒	A statement on whether measurements were taken from distinct samples or whether the same sample was measured repeatedly
☐	☒	The statistical test(s) used AND whether they are one- or two-sided<br>*Only common tests should be described solely by name; describe more complex techniques in the Methods section.*
☒	☐	A description of all covariates tested
☐	☒	A description of any assumptions or corrections, such as tests of normality and adjustment for multiple comparisons
☐	☒	A full description of the statistical parameters including central tendency (e.g. means) or other basic estimates (e.g. regression coefficient) AND variation (e.g. standard deviation) or associated estimates of uncertainty (e.g. confidence intervals)
☐	☒	For null hypothesis testing, the test statistic (e.g. *F*, *t*, *r*) with confidence intervals, effect sizes, degrees of freedom and *P* value noted<br>*Give P values as exact values whenever suitable.*
☒	☐	For Bayesian analysis, information on the choice of priors and Markov chain Monte Carlo settings
☒	☐	For hierarchical and complex designs, identification of the appropriate level for tests and full reporting of outcomes
☐	☒	Estimates of effect sizes (e.g. Cohen's *d*, Pearson's *r*), indicating how they were calculated

Our web collection on statistics for biologists contains articles on many of the points above.

Software and code

Policy information about availability of computer code

Data collection	No software was used for data collection.
Data analysis	The implementation of CEFCON is available at https://github.com/WPZgithub/CEFCON. CEFCON was developed in Python 3.8 mainly with the following package dependencies: pytorch (v1.8.0), torch-geometric (v2.1.0), scanpy (v1.8.2), networkx (v2.8.0), cvxpy (v1.2.0), gurobipy (v9.5.0), pyscenic (v0.12.0), rpy2 (v3.4.1), matplotlib-venn (v0.11.7) and palantir (v1.0.1). The AUPRC for evaluating the GRN construction is calculated through the PRROC package in a R (v3.6) environment.

For manuscripts utilizing custom algorithms or software that are central to the research but not yet described in published literature, software must be made available to editors and reviewers. We strongly encourage code deposition in a community repository (e.g. GitHub). See the Nature Portfolio guidelines for submitting code & software for further information.

Data

Policy information about availability of data

All manuscripts must include a data availability statement. This statement should provide the following information, where applicable:

- Accession codes, unique identifiers, or web links for publicly available datasets
- A description of any restrictions on data availability
- For clinical datasets or third party data, please ensure that the statement adheres to our policy

All the datasets analyzed in this study are publicly available. The prior gene interaction network was from NicheNet, which can be downloaded from https://github.com/saeyslab/nichenetr. The scRNA-seq datasets are available in the Gene Expression Omnibus (GEO) under accession codes: GSE75748 [https://

www.ncbi.nlm.nih.gov/geo/query/acc.cgi?acc=GSE75748] (hESC), GSE81252 [https://www.ncbi.nlm.nih.gov/geo/query/acc.cgi?acc=GSE81252] (hHep), GSE98664 [https://www.ncbi.nlm.nih.gov/geo/query/acc.cgi?acc=GSE98664] (mESC), GSE48968 [https://www.ncbi.nlm.nih.gov/geo/query/acc.cgi?acc=GSE48968] (mDC) and GSE81682 [https://www.ncbi.nlm.nih.gov/geo/query/acc.cgi?acc=GSE81682] (mHSC). The ChIP-seq and loss-of-function/gain-of-function (lof/gof) data for validating the constructed GRN were obtained from BEELINE [21]. The gene expression response data after the forced induction of TFs in mESCs for validating the constructed GRN are available in the GEO database under accession code GSE31381 [https://www.ncbi.nlm.nih.gov/geo/query/acc.cgi?acc=GSE31381]. The TF list of human and mouse for GRN evaluation and analyses are available at the GitHub of pySCENIC (https://github.com/aertslab/pySCENIC/tree/master/resources). The three GO gene sets, including GO:0045165 [https://www.informatics.jax.org/go/term/GO:0045165], GO:0019827 [https://www.informatics.jax.org/go/term/GO:0019827] and GO:0007492 [https://www.informatics.jax.org/go/term/GO:0007492], for evaluating the identified driver regulators are available at http://www.informatics.jax.org/vocab/gene_ontology. The lists of the literature-curated key regulators about ESC for both human and mouse were directly obtained from the pubications with DOI https://doi.org/10.1530/REP-07-0359 and https://doi.org/10.1016/j.cell.2011.01.032. All the data used in this study are available at Zenodo (https://doi.org/10.5281/zenodo.7564872).

Human research participants

Policy information about studies involving human research participants and Sex and Gender in Research.

Reporting on sex and gender	N/A.
Population characteristics	N/A.
Recruitment	N/A.
Ethics oversight	N/A.

Note that full information on the approval of the study protocol must also be provided in the manuscript.

Field-specific reporting

Please select the one below that is the best fit for your research. If you are not sure, read the appropriate sections before making your selection.

☒ Life sciences ☐ Behavioural & social sciences ☐ Ecological, evolutionary & environmental sciences

For a reference copy of the document with all sections, see nature.com/documents/nr-reporting-summary-flat.pdf

Life sciences study design

All studies must disclose on these points even when the disclosure is negative.

Sample size	No statistical method was used to predetermine sample size. We used published datasets, and thus the the sample sizes in this study are almost the same as in the original studies, except for the difference in data preprocessing. For each analysis, the sample size was sufficient to derive statistically meaningful results.
Data exclusions	We excluded low expression genes and low quality cells, and we chose highly variable genes in the experiments, as described in the Method section. More specifically,<br>1) for all the benchmarking experiments, we chose 1,000 highly variable genes;<br>2) for the in-depth analyses of mouse hematopoietic stem cell differentiation, the cells with more than 200 zero expressed genes were deleted and the genes expressed on fewer than 5 cells were removed. The top 3,000 highly variable genes were selected using the 'cell_ranger' method in the SCANPY package.
Replication	We run all the algorithms 20 replications with random initial seeds, and average of the values was taken for the analyses. We provide the code necessary for replicating the results. Different package versions or computational environments might lead to slightly different outputs.
Randomization	No randomization was conducted in our study. Our model is unsupervised and the learning process dose not need to randomly split train/test set.
Blinding	All results are based on published data which have been studied in their original publications. Therefore, blinding from investigators is not possible when we reanalyzed the data. Group allocation information was never provided to the computational algorithms.

Reporting for specific materials, systems and methods

We require information from authors about some types of materials, experimental systems and methods used in many studies. Here, indicate whether each material, system or method listed is relevant to your study. If you are not sure if a list item applies to your research, read the appropriate section before selecting a response.

Materials & experimental systems

n/a	Involved in the study
☒	☐ Antibodies
☒	☐ Eukaryotic cell lines
☒	☐ Palaeontology and archaeology
☒	☐ Animals and other organisms
☒	☐ Clinical data
☒	☐ Dual use research of concern

Methods

n/a	Involved in the study
☒	☐ ChIP-seq
☒	☐ Flow cytometry
☒	☐ MRI-based neuroimaging
